# A Soybean Resistant Protein-Containing Diet Increased the Production of Reg3γ Through the Regulation of the Gut Microbiota and Enhanced the Intestinal Barrier Function in Mice

**DOI:** 10.3389/fnut.2021.701466

**Published:** 2021-08-19

**Authors:** Tasuku Ogita, Fu Namai, Ayane Mikami, Takahiro Ishiguro, Koji Umezawa, Yutaka Uyeno, Takeshi Shimosato

**Affiliations:** ^1^Department of Biomolecular Innovation, Institute for Biomedical Sciences, Shinshu University, Nagano, Japan; ^2^Department of Pathology, Graduate School of Biomedical Sciences, Nagasaki University, Nagasaki, Japan; ^3^Food Research Center, Asahimatsu Foods Co., Ltd., Nagano, Japan; ^4^Faculty of Agriculture, Shinshu University, Nagano, Japan

**Keywords:** cecal microbiota, intestinal barrier, PiCRUSt, Reg3γ, soybean resistant protein

## Abstract

The maintenance of intestinal homeostasis is necessary for a good quality of life, and strengthening of the intestinal barrier function is thus an important issue. Therefore, we focused on soybean resistant protein (SRP) derived from *kori-tofu* (freeze-dried tofu), which is a traditional Japanese food, as a functional food component. In this study, to investigate the effect of SRP on the intestinal barrier function and intestinal microbiota, we conducted an SRP free intake experiment in mice. Results showed that ingestion of SRP decreased the serum level of lipopolysaccharide-binding protein and induced the expression of Reg3γ, thereby improving the intestinal barrier function. In addition, SRP intake induced changes in the cecal microbiota, as observed by changes in β-diversity. In particular, in the microbiota, the up-regulation of functional gene pathways related to the bacterial invasion of epithelial cells (ko05100) was observed, suggesting that Reg3γ expression was induced by the direct stimulation of epithelial cells. The results of this study suggest that SRP is a functional food component that may contribute to the maintenance of intestinal homeostasis.

## Introduction

The gastrointestinal tract is a frontline barrier against external factors, such as food antigens and microbes. Intestinal epithelial cells (IECs), immune cells, and the microbiota are related to each other, and together, they help maintain intestinal homeostasis (1). Disruption of the intestinal system is involved in various disorders, such as obesity and inflammatory bowel disease (2, 3). The intestinal barrier is classified into physical and chemical barriers, and it enables intestinal homeostasis to be maintained by separating the non-self and the host (4). The physical barrier consists of mucus and the firm linking of IECs, i.e., tight junctions. In contrast, the chemical barrier consists of antimicrobial peptides (5). In particular, the regenerating islet-derived 3 (Reg3) protein, which is a C-type lectin, plays an essential role in the segregation of the microbiota and IECs mainly *via* the sterilization of Gram-positive bacteria by binding to the microbial cell surface and perforating it (6, 7). It is known that Reg3γ is induced by the interleukin (IL)-22/signal transducer and activator of transcription 3 (STAT3) pathway (8), and IL-22 is mainly secreted by the type 3 innate lymphoid cells (ILC3) stimulated by microbiota (9). In addition, Reg3γ can be regulated by bacterial ligands through Toll like receptor (TLR)/Myd88 signals (10).

The enhancement and/or improvement of these barrier functions are important in achieving a better quality of life, and strengthening of the intestinal barrier function through the diet is an ideal strategy as it is generally safe and cost-efficient. In this context, we focused on resistant proteins (RPs) as functional food components. RPs are protein remnants or indigestible protein complexes that have physiological functions similar to dietary fibers (11). Several studies on the functional and health attributes of RPs have been conducted, and it has been reported that the feeding of RPs to rats increased the levels of immunoglobulin A and mucin, which are associated with the intestinal barrier (12, 13). In these studies, it was also pointed out that the intestinal microbiota that metabolizes the RPs plays an important role in the functional effects of the RPs.

Freeze-dried tofu, a traditional Japanese food that is also known as *kori-tofu*, contains many soybean resistant proteins (SRP) due to its characteristic manufacturing process involving freezing, thawing, and drying (14, 15). The intake of *kori-tofu* itself or the intake of SRP from *kori-tofu* improved the metabolism of lipids and sugars. Takahashi and Konishi reported that the feeding of *kori-tofu* to rats for 3 weeks reduced the expression of genes related to lipogenesis, and resulted in a lower serum lipid level (16). In addition, Sugano et al. revealed that SRP intake decreased the serum cholesterol level in *in vivo* experiments in rats (17). Although there have been several studies on the effects of SRP on metabolism, no studies have examined the effects of SRP on the intestinal barrier function and the intestinal microbiota. The purpose of the present study was to investigate the effects of SRP intake on the intestinal barrier function and the cecal microbiota in mice to further determine the functionality of SRP.

## Materials and Methods

### Preparation of the SRP

SRP was prepared according to the method of Nishimura et al. with minor modifications (18). Briefly, 1 g of pepsin (FUJIFILM Wako Pure Chemical Co., Ltd., Tokyo, Japan) was combined with 100 g of soy protein (Fujipro E, Fuji Oil Co., Ltd., Osaka, Japan) suspended in distilled water; the mixture was adjusted to pH 1.9 with HCl, and incubated at 37°C for 5 h with shaking. Next, the pH was adjusted to 7.2 with NaOH, then 3 g of pancreatin (FUJIFILM Wako Pure Chemical Co., Ltd.) was added to the mixture, which was incubated with shaking at 37°C for another 14 h. Following this digestion, the enzyme was inactivated by heat treatment at 80°C for 10 min. The pH was adjusted to 4.5–5.0 with HCl, then the mixture was centrifuged at 9,000 × *g* for 15 min. The pellet was washed three times with distilled water, then centrifuged at 9,000 × *g* for 15 min, and the resulting pellet was lyophilized. The SRP was incorporated into the diet as described below.

The composition of SRP was analyzed by following the standard AOAC methods with some modifications (Table 1). Briefly, the moisture content was determined by constant weight drying in an oven at 105°C. The protein content (N ×6.25) was measured by the Kjeldahl method using a Kjeldahl system (Buchi K-426 and K-350, BUCHI, Switzerland). The lipid content was measured by n-hexane 2-propanol mixture (3:2) extraction using a Soxhlet system (Buchi E-816, BUCHI, Switzerland). The ash content was determined by incineration in a muffle furnace at 550°C for 6 h. The remaining percentage was taken to be the carbohydrate content.

**Table 1:** The composition of SRP.

**Ingredients**	**Composition (%)**
Moisture	5.1
Protein	70.1
Lipid	1.1
Ash	5.2
Carbohydrate	18.5

### Mice

Female C57BL/6 mice (5 weeks of age) were purchased from Japan SLC (Hamamatsu, Japan) and housed in standard plastic cages in a temperature-controlled (24 ± 1°C) room with a 12-/12-h light/dark cycle throughout the study.

### SRP-Feeding Experiment

**Figure 1:**
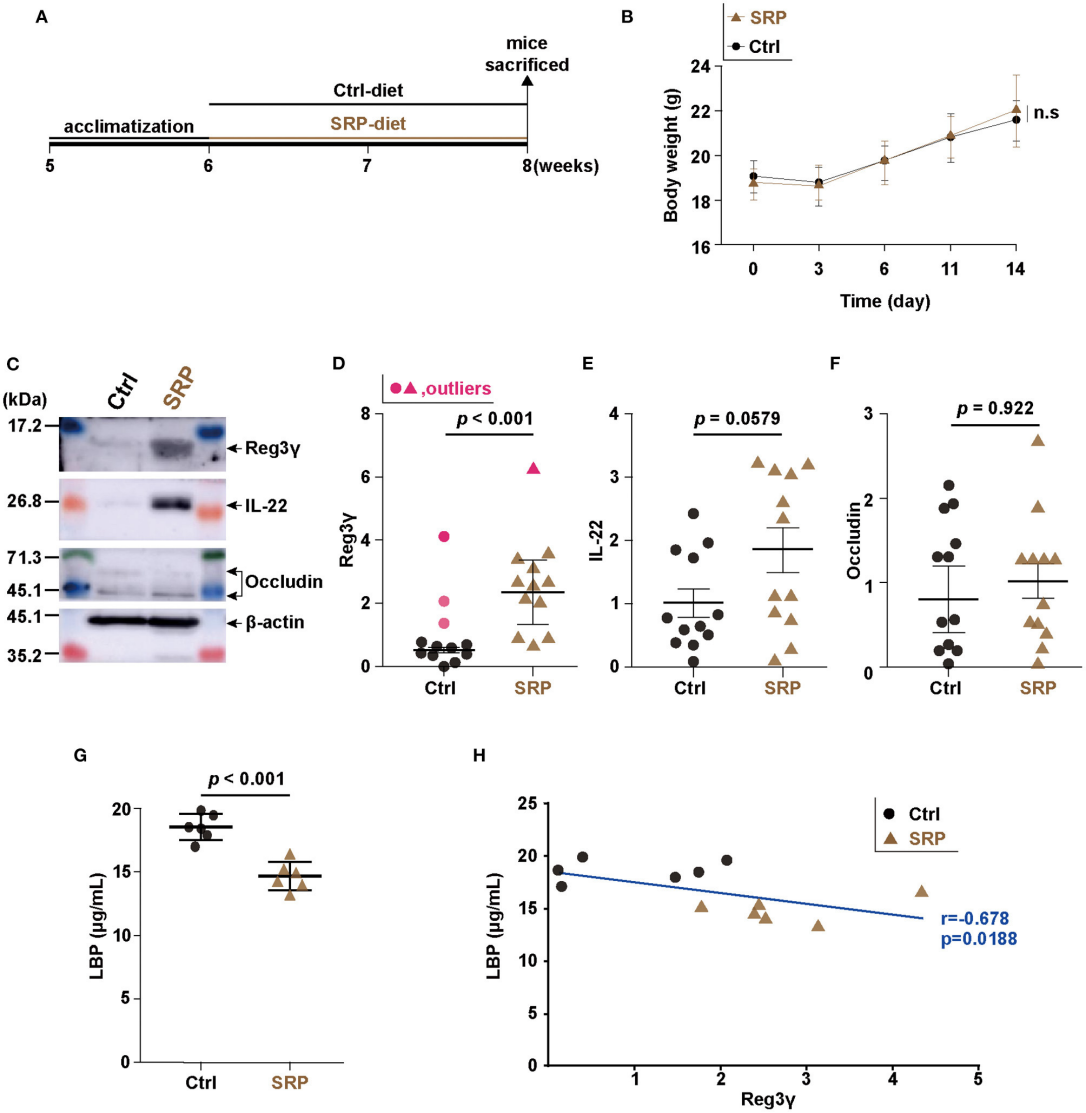
Changes in intestinal barrier function due to soybean resistant protein (SRP)-diet intake. Schedule for the experiment examining the effects of *ad libitum* feeding of the SRP-diet or the control diet **(A)**. The mice were weighed every 3–5 days during the experimental period **(B)**. Representative western blots **(C)** and densitometory analyses **(D–F)**. The expression of Reg3γ **(D)**, IL-22 **(E)**, and occludin **(F)** in cecal tissue was assessed by western blotting [*n* = 9–12; values are expressed as the mean (SD)]. The LBP concentration was measured by ELISA [**G**; *n* = 6; values are expressed as the mean (SD)]. The correlation between the production of Reg3γ and LBP (**H**; *n* = 6). The correlation was assessed by Pearson's correlation coefficient. *p* indicates the *p*-value. Statistical analysis was performed after the omission of outliers. Ctrl, control diet group; n.s, not significant; SRP, soybean resistant protein diet group; LBP, lipopolysaccharide-binding protein; Reg3γ, regenerating islet-derived protein 3 gamma; IL-22, interleukin 22; SD, standard deviation; ELISA, enzyme-linked immunosorbent assay.

The SRP-feeding experiment was performed according to the schedule shown in Figure 1A. Mice were acclimatized for 1 week, then randomly allocated into two groups, the SRP group or the Control (Ctr) group. Then mice were given *ad libitum* access to an SRP-containing diet (SRP-diet; AIN-93G + 5% SRP, Oriental Yeast Co., Tokyo, Japan) or a control diet (AIN-93G, Oriental Yeast), respectively, for 2 weeks. The animals also had *ad libitum* access to tap water and were weighed every 3–5 days during the experimental period. After 2 weeks of feeding of the SRP-diet or control diet, the mice were sacrificed, and the blood and cecum were collected. The cecum was removed from each animal at necropsy by locating and excising (with scissors) the pouch while avoiding the segments connecting to the adjacent small intestine and proximal colon. The excised tissue was sliced open, and the cecal contents were placed in a tube containing RNAlater (Thermo Fisher Scientific, Waltham, MA, USA). After 24 h of treatment with RNAlater, the cecal contents were separated from the RNAlater by centrifugation at 8,000 × *g* for 5 min, then stored at −20°C until DNA extraction. Following the removal of the cecal contents, the cecal tissue itself was rinsed with physiological saline to remove any remaining contents, then flash-frozen at −80°C for subsequent protein isolation and western blotting.

### Western Blotting

The flash-frozen cecal tissue samples were suspended in lysis buffer (1% sodium dodecyl sulfate, 1% Triton X-100, 1% sodium deoxycholate, and 30 mM Tris, pH 7.4). Then, the lysates were suspended in sample buffer (FUJIFILM Wako Pure Chemical Co., Ltd.) and incubated at 95°C for 10 min. Samples were resolved by 15% (v/v) sodium dodecyl sulfate polyacrylamide gel electrophoresis. Proteins were then transferred from the gel onto a Hybond-P PVDF membrane (GE Healthcare Japan, Tokyo, Japan). After blocking for 1 h, the membrane was incubated overnight with anti-mouse Reg3γ (SAB4301004), anti-mouse β-actin (clone AC-15), anti-mouse occludin (clone OC-3F10; Sigma-Aldrich, St. Louis, MO, USA), or anti-mouse IL-22 (clone BL35175; BioLegend, San Diego, CA, USA) antibody at 4°C, followed by incubation with the secondary antibody (HRP Goat anti-rat IgG antibody, clone Poly4054; BioLegend). The immunoblots were visualized using ECL Prime Western Blotting Detection Reagent (GE Healthcare Japan). The protein bands were detected using ImageQuant LAS 500 (GE Healthcare Japan), and analyzed using ImageJ software (Version 1.51; National Institutes of Health, Bethesda, MD, USA). Protein quantitation was performed by densitometry; the values were normalized to the expression level of the housekeeping protein β-actin.

### Enzyme-Linked Immunosorbent Assay

The collected blood was incubated for 30 min at 25°C, then incubated overnight at 4°C. The coagulated blood was removed by centrifugation (1,000 × *g*, 4°C, 30 min) to obtain the serum. A commercially available ELISA kit was used to quantify the lipopolysaccharide-binding protein (LBP; Biometec, Greifswald, Germany) in the serum according to the manufacturer's instructions.

### 16S rRNA V3-V4 Sequence Analysis

An aliquot (5–10 mg) of each cecal content sample was mixed with the Inhibitory Ex buffer (200 μL) of the QIAamp Fast DNA Stool Mini Kit (Qiagen, Germantown, MD, USA) in a Zirco Prep Mini tube (Nippon Genetics Co., Ltd., Tokyo, Japan). The sample was shaken for 5 min using a Bug Crasher μT-12 (Taitec Co., Saitama, Japan). DNA was purified from the mixture using the QIAamp Fast DNA Stool Mini Kit (Qiagen). PCR was performed using a 25-μL reaction volume containing 12.5 μL of 2 × Gflex PCR buffer, 0.5 μL of Tks Gflex DNA polymerase (TaKaRa Bio, Inc., Shiga, Japan), 1 μL each of 10 μM 1st PCR primers (341F and 806R, 16S rRNA gene V3-V4 regions), and 2 μL of cecal DNA. PCR was performed using the following program: 1 cycle at 94°C for 1 min, followed by 28 cycles at 98°C for 10 s, 50°C for 15 s, and 68°C for 15 s. Next, index PCR was performed using a 25-μL reaction volume containing 12.5 μL of 2 × Gflex PCR buffer, 0.5 μL of Tks Gflex DNA polymerase, 1 μL each of Nextera XT Index primers (Illumina, Inc., San Diego, CA, USA), and 2 μL of the first-round PCR product. Index PCR was performed using the following program: 1 cycle at 94°C for 1 min, followed by 8 cycles at 98°C for 10 s, 60°C for 15 s, and 68°C for 15 s. After the index PCR reactions, the products were purified with the Fast Gene Gel/PCR Extraction Kit (Nippon Genetics Co., Ltd.), and quantified using Quant-iT (Life Technologies Japan, Tokyo, Japan). Equivalent quantities of PCR products from each sample were pooled and sent to TaKaRa Bio. Paired-end sequencing (250 bp) of the purified PCR products was performed using MiSeq (Illumina, Inc.). The resulting sequence files, fastq files, were uploaded to DDBJ (Accession Number: PRJDB11968). Paired reads were joined by join_paired_ends using the relevant script in the QIIME 1.9.1 pipeline, an application consistent with the use of this script in similar previous studies (19). Notably, the default settings were used for “Minimum allowed overlap in base-pairs required to join pairs” and “Maximum allowed % differences within region of overlap.” The sequencing data were analyzed using the QIIME 1.9.1 pipeline, including chimera checking and the α-/β-diversity (19). The reads from all of the samples were clustered into operational taxonomic units (OTUs). OTU clustering was performed using the Greengenes database (gg_13_5; http://greengenes.lbl.gov/Download/) (19). We clustered sequences based on a cut-off of 97% sequence identity to the respective reference sequences. The data for species-level taxonomy were obtained by filtering the OTU tables containing taxonomic data generated by the RDP Classifier (20) at the genus level. Representative sequences were then extracted, and species-level matches within the National Center for Biotechnology Information database were identified using BLAST. The minimum percent query coverage of an alignment was 97%. The setting for the alignments per read was “1” (default setting). The setting for the sequence similarity threshold was 97% sequencing identity (default setting). When multiple best hits with differing taxonomies were obtained, we considered both the E value and sequence identity. The α-diversity index was measured using Chao1, Shannon index, phylogenetic diversity, and observed OTU analyses, while β-diversity analysis was performed using weighted and unweighted UniFrac. The characteristics of the cecal microbiota were determined as the linear discriminant analysis (LDA) score by Linear discriminant analysis Effect Size (LEfSe) version 1.0 in the Huttenhower Lab Galaxy server (21).

### Prediction of Cecal Microbiota Metagenomes Using PICRUSt and HUMAnN2

Cecal microbiota metagenomes were predicted from the bacterial 16S rRNA gene by the PICRUSt (Version 1.0.0) pipeline (22). We obtained the closed-reference OTU from 16S rRNA gene data with the QIIME 1.9.1 pipeline using the Greengenes database (gg_13_5). The OTU table was normalized to the 16S rRNA copy number. Metagenomes were predicted from the Kyoto Encyclopedia of Genes and Genomes database. The metagenome pathways were created by the HUMAnN2 (Version 0.7.1) pipeline (23). The characteristics of the metagenomes were determined as the LDA score from LEfSe version 1.0 in the Huttenhower Lab Galaxy server (21).

### Statistical Analysis

The R software package (Version 3.3.1) was used for all statistical analyses. Outliers were identified using the Smirnov-Grubbs test and omitted prior to further statistical analysis. Normality of the variances was confirmed using the Shapiro-Wilk normality test. The statistical differences of non-normal data sets were determined by the Wilcoxon rank sum test. The *F*-test was used for analyzing the homogeneity of variances or homoscedasticity. The two-tailed *t*-test or Welch's *t*-test (for data with or without homoscedasticity, respectively) was used to compare the SRP group with the Ctrl group. Pearson's correlation coefficient (with normality) was calculated for linear and correlation analyses. A *p*-value of <0.05 was considered to be statistically significant.

## Results

### The SRP-Diet Induced Reg3γ Expression in the Cecum and Suppressed the Serum LBP Concentration

In the SRP-diet feeding experiment, the body weight did not differ significantly between the SRP and Ctrl groups (Figure 1B). Western blotting of Reg3γ, IL-22, and occludin from cecal tissue showed that the Reg3γ protein level was higher in the SRP group than in the Ctrl group (Figures 1C,D), while the levels of IL-22 and occludin proteins did not differ significantly between the two groups (Figures 1C,E,F). The serum LBP concentration was significantly lower in the SRP group than in the Ctrl group (Figure 1G). In addition, the serum LBP concentration showed a significant positive correlation with the Reg3γ protein level in cecal tissue (*r* = −0.678, *p* = 0.0188; Figure 1H).

### The SRP-Diet Induced Changes in the Cecal Microbiota

**Figure 2:**
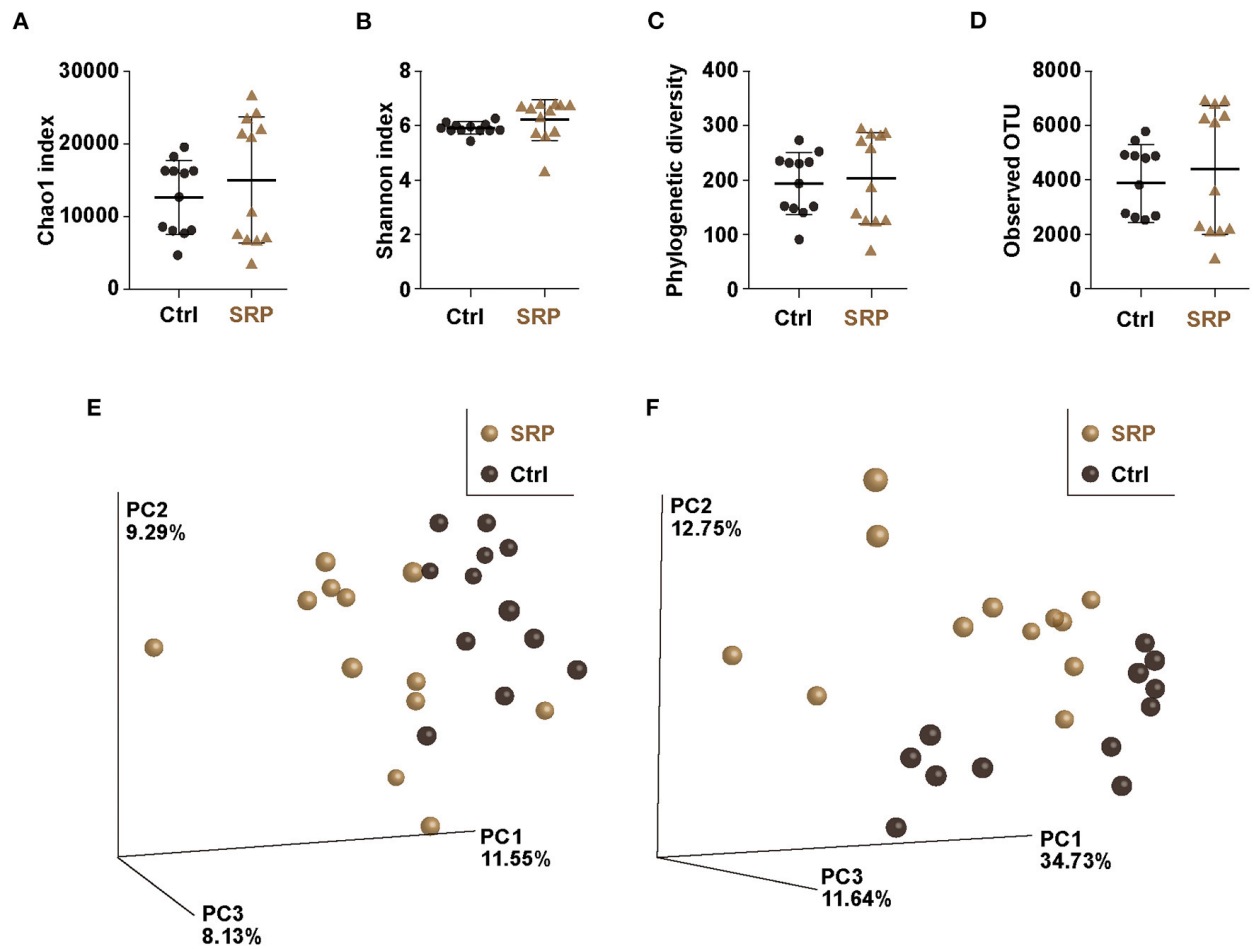
The α- and β-diversity indices of the cecal microbiota. The Chao1 index **(A)**, Shannon index **(B)**, phylogenetic diversity **(C)**, and observed OTUs **(D)** were calculated for the Ctrl and SRP groups [*n* = 12; values are expressed as the mean (SD)]. β-Diversity by weighted **(E)** and unweighted **(F)** UniFrac was assessed as the distance metric between the Ctrl and SRP groups (*n* = 12).

Considering that RPs can be assimilated by microbiota, the effect of the SRP-diet on the cecal microbiota was analyzed using a next-generation sequencing approach based on the 16S rRNA V3-V4 sequences. Diversity analysis revealed no significance changes in the **α**-diversity (Chao1, phylogenetic diversity whole tree, Shannon index, and observed OTU analyses) as a result of the SRP-diet (Figures 2A–D). In contrast, principal component analysis of the β-diversity showed the formation of different clusters in the SRP and Ctrl groups, suggesting that the structure of the microbiota was changed by the SRP-diet (Figures 2E,F).

**Figure 3:**
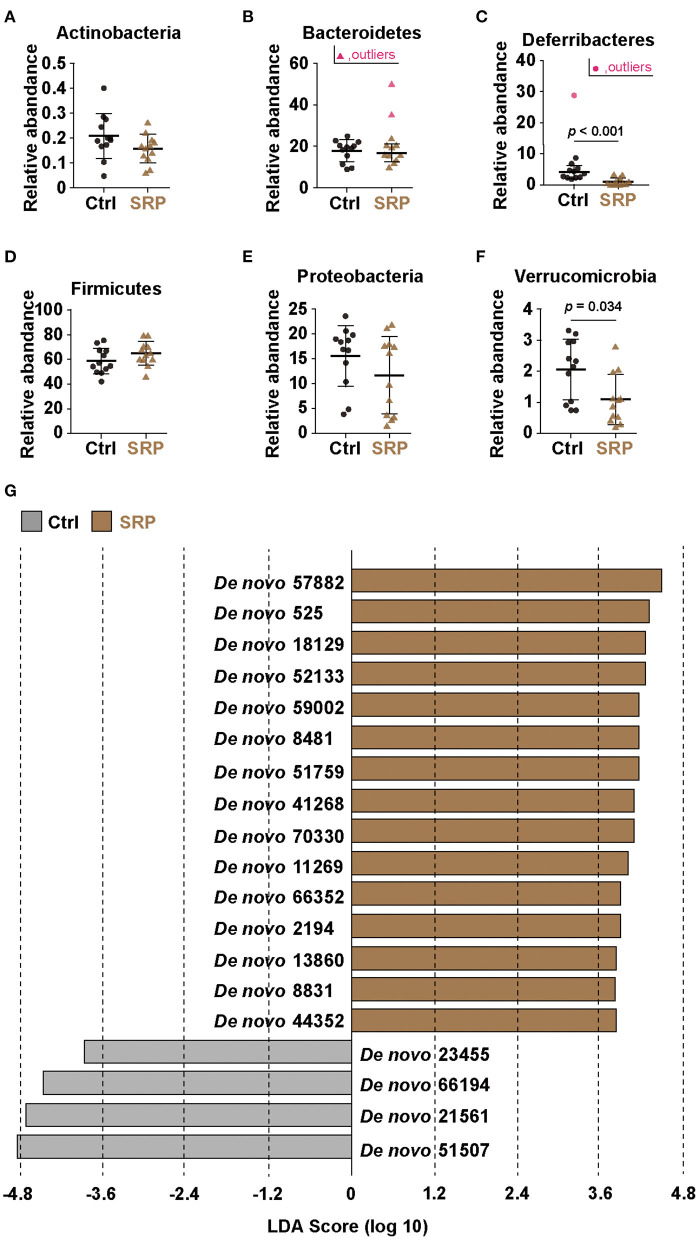
Effects of the SRP-diet on the cecal microbiota. Proportions of the total content of Actinobacteria **(A)**, Bacteroidetes **(B)**, Deferribacteres **(C)**, Firmicutes **(D)**, Proteobacteria **(E)**, and Verrucomicrobia **(F)** were assessed in the cecal contents by MiSeq [*n* = 10–12; values are expressed as the mean (SD)]. *p* indicates the *p*-value. Selection of the cecal microbiota was based on linear discriminant analysis (LDA) **(G)**. Operational taxonomic units (OTUs) were determined to be significantly different according to the LDA score (LDA > 3). OTUs showing significant differences (*p* < 0.05) between the two groups were extracted by the Kruskal-Wallis and Wilcoxon rank-sum tests. Ctrl, control diet group; SRP, soybean resistant protein diet group.

The proportions of the phyla Actinobacteria, Bacteroidetes, Firmicutes, and Proteobacteria detected did not differ between the SRP and Ctrl groups (Figures 3A,B,D,E). In contrast, the proportions of the phyla Deferribacteres and Verrucomicrobia detected were significantly lower in the SRP group than in the Ctrl group (Figures 3C,F). In addition, significant changes were observed at the species level. OTUs with a *p*-value < 0.05, relative abundance > 1%, and LDA score > 3 in the SRP and Ctrl groups are shown in Figure 3G; among these OTUs, the 15 OTUs of the SRP group included Bacteroidetes (14 OTUs) and Firmicutes (1 OTU; Figure 3G). Representative sequences from the SRP and Ctrl groups were analyzed using BLAST to identify species-level matches within the database (Table 2).

**Table 2:** Characteristics of 19 OTUs in the cecal contents.

**OTU No**.	**Accession No**.	**Taxonomy**	**Identity (%)**	***E*-value**
*De novo* 525	KR364784.1	*Muribaculum intestinale* strain YL27	88.72	1.09E-170
*De novo* 2194	NR_133742.1	*Falsiporphyromonas endometrii* strain LMM 40	96.33	2.69E-45
*De novo* 8481	NR_118219.1	*Alistipes senegalensis* JC50	93.289	7.32E-59
*De novo* 11269	KR364784.1	*Muribaculum intestinale* strain YL27	91.342	0.0
*De novo* 13860	NR_113152.1	*Alistipes putredinis* strain JCM 16772	95.455	0.0
*De novo* 18129	KR364784.1	*Muribaculum intestinale* strain YL27	88.223	5.61E-168
*De novo* 21561	LC515638.1	*Clostridium celatum* G085	98.64	0.0
*De novo* 23455	MN081648.1	*Parabacteroides distasonis* strain WYJ14_D4	99.13	0.0
*De novo* 41268	KR364784.1	*Muribaculum intestinale* strain YL27	91.54	0.0
*De novo* 44352	KR364784.1	*Muribaculum intestinale* strain YL27	92.873	0.0
*De novo* 51507	NR_042896.1	*Mucispirillum schaedleri* strain HRI I17	99.00	0.0
*De novo* 51759	KR364784.1	*Muribaculum intestinale* strain YL27	89.20	8E-173
*De novo* 52133	KR364784.1	*Muribaculum intestinale* strain YL27	90.24	7E-180
*De novo* 57882	NR_144611.1	*Flintibacter butyricus* strain BLS21	99.099	0.0
*De novo* 58822	NR_074436.1	*Akkermansia muciniphila* strain ATCC BAA-835	99.00	0.0
*De novo* 59002	NR_113152.1	*Alistipes putredinis* strain JCM 16772	95.445	0.0
*De novo* 66194	KF698402.1	*Bacterium* WCE2004	88.82	2E-156
*De novo* 66352	NR_113152.1	*Alistipes putredinis* strain JCM 16772	95.302	1.16E-62
*De novo* 70330	KR364784.1	*Muribaculum intestinale* strain YL27	91.76	0.0

### Correlation Analysis of Reg3γ Expression and the OTUs

**Figure 4:**
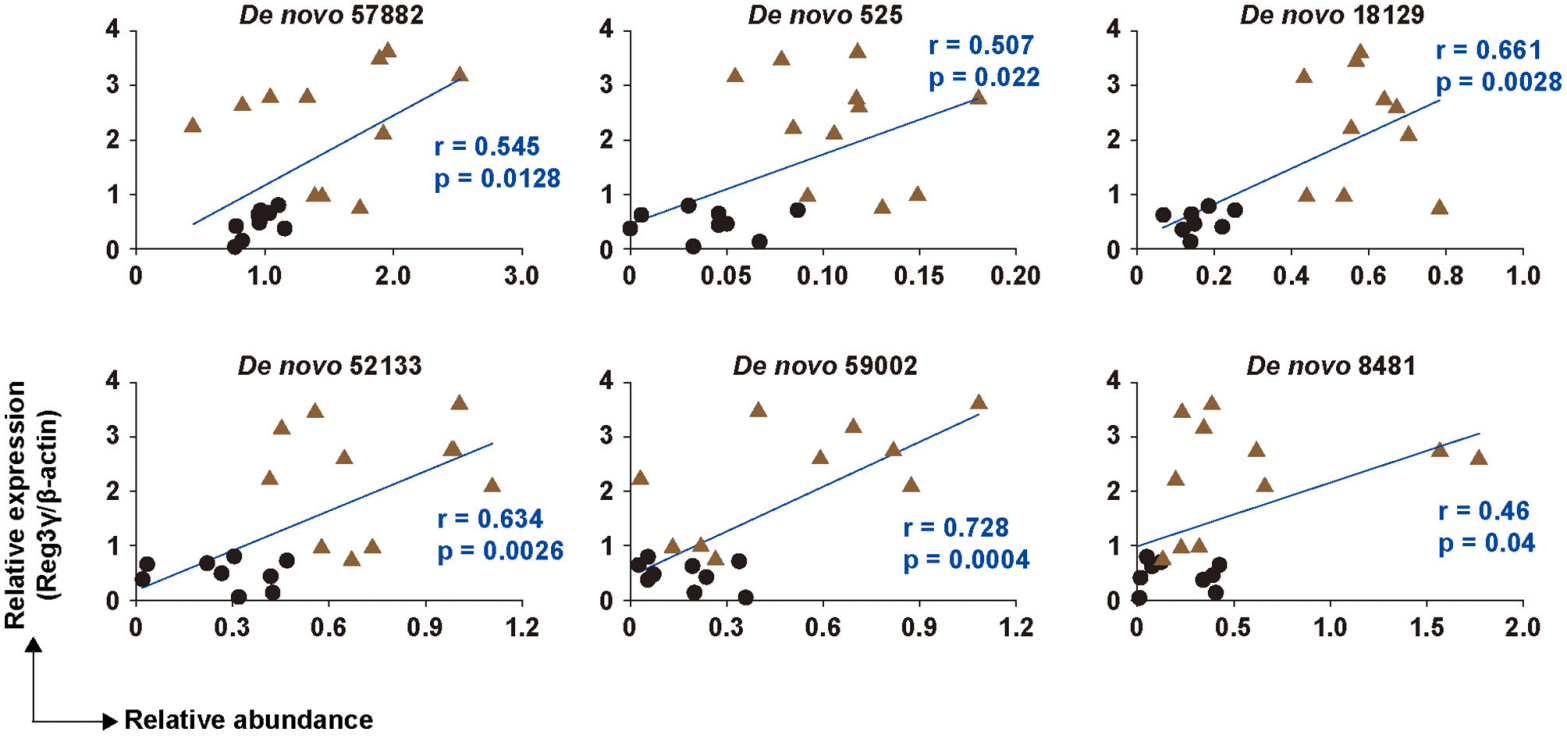
Correlation between six OTUs and the production of Reg3γ in cecal tissue. The correlation between the production of Reg3γ and the abundance of *De Novo* 57,882, 525, 18,129, 52,133, 59,002, and 8,481 (*n* = 6–12). The correlation was assessed by Pearson's correlation coefficient. *p* indicates the *p*-value. Statistical analysis was performed after the omission of outliers.

To investigate the relationship between the microbiota and Reg3γ expression, we analyzed the correlation between the six bacterial OTUs that changed the most at the species level and the expression level of Reg3γ. Results showed a significant positive correlation between the Reg3γ expression level and each of the six bacterial OTUs (Figure 4). Furthermore, the correlation coefficient showed that *De novo* 59002 (*Alistipes putredinis*) had a strong correlation with Reg3γ expression, and *De novo* 57882 (*Flintibacter butyricus*), *De novo* 525 (*Muribaculum intestinale*), *De novo* 18129 (*M. intestinale*), and *De novo* 52133 (*M. intestinale*) had a moderate correlation with Reg3γ expression (Figure 4).

### Prediction of the Functional Potential of the Microbiota in Mice Fed the SRP-Diet

**Figure 5:**
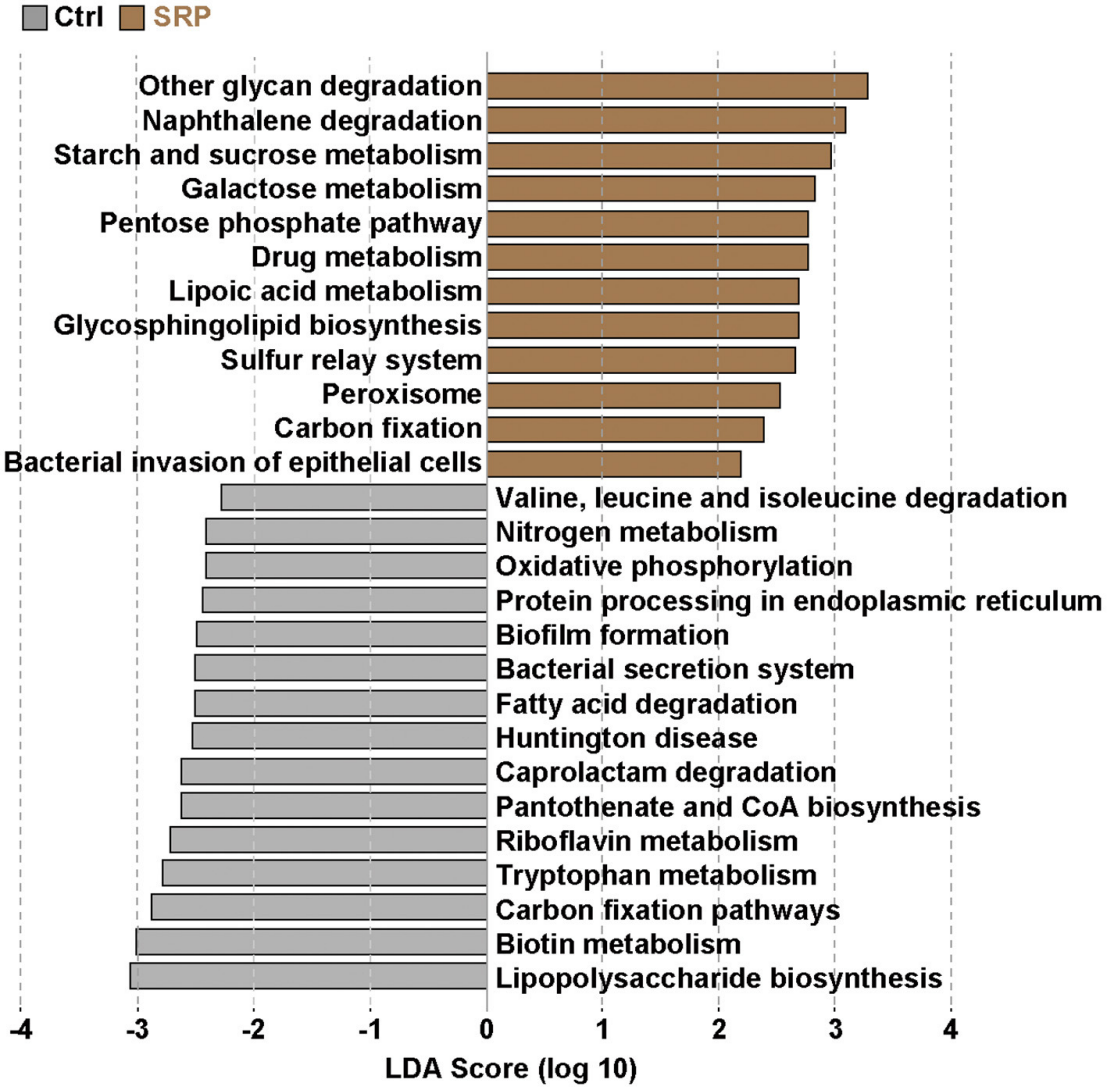
Prediction of functional gene pathways of the cecal microbiota. The changes in the functional gene pathways in the cecal microbiota due to the SRP-diet were predicted using PICRUSt analysis. Brown bars represent the up-regulated pathways in the SRP group. In contrast, gray bars indicate the down-regulated pathways in the SRP group. Pathways were determined to be significantly different according to the LDA score (LDA > 2). Pathways showing significant differences (*p* < 0.05) between the two groups were extracted by the Kruskal-Wallis and Wilcoxon rank-sum tests. Ctrl, control diet group; SRP, soybean resistant protein diet group.

Since there is a relationship between microorganisms and their functional genes (24), we analyzed the functional attributes of the microbiome using PICRUSt. It was confirmed that the functional gene pathways involved in carbohydrate metabolism, such as other glycan degradation (ko00511), starch and sucrose metabolism (ko00500), and galactose metabolism (ko00052), were up-regulated in the SRP group when compared to the Ctrl group (Figure 5). In addition, the pathway involved in the bacterial invasion of epithelial cells (ko05100) was also up-regulated in the SRP group (Figure 5). In contrast, the functional gene pathway involved in lipopolysaccharide biosynthesis (ko00540) was down-regulated in the SRP group when compared to the Ctrl group.

## Discussion

In this study, we assessed the functionality of SRP derived from *kori-tofu* in mice with the aim of enhancing intestinal homeostasis through the use of functional food components. Free intake of feed containing SRP increased the expression of the antibacterial peptide Reg3γ in the cecum, suggesting that the intestinal barrier function, especially the chemical barrier, was enhanced in the mice. Indeed, the blood LBP level, a major marker of intestinal barrier failure (25–27), was significantly decreased in the SRP group. This indicated that SRP intake suppressed the invasion of microbes from the intestine into the body. Antibacterial peptides enhance the physical separation of gut bacteria from the host, which is primarily due to the bactericidal effect (28). Furthermore, Reg3γ is known not only for its antibacterial effect, but also for its ability to suppress apoptosis in epithelial cells (29). In fact, Reg3γ-deficient mice have been shown to have increased contact between epithelial cells and gut bacteria, facilitating their translocation into tissues (30). These results suggest that the induction of Reg3γ expression by SRP strengthened the separation of the intestinal microbiota and the host, resulting in a decrease in blood LBP. In contrast, there was no change in the expression levels of IL-22, a Reg3γ-inducing cytokine (31), and occludin, a tight junction protein (32). This suggests that Reg3γ is induced by pathways other than the IL-22/STAT3 pathway, and that the SRP-induced enhancement of antibacterial peptides, rather than tight junctions, plays an important role in maintaining intestinal homeostasis. However, further research is necessary to support this hypothesis because tight junction proteins other than occludin were not measured in this study and the SRP group may be divided into high and low IL-22-expressing subgroups.

Given that RPs can circumvent the digestive process and are metabolized by intestinal microbiota (11) and that the intestinal microbiota is important for Reg3γ expression (33), the intestinal microbiota was analyzed based on 16S rRNA sequence analysis. The results showed no significant change in α-diversity, but β-diversity formed different clusters in the SRP and Crtl groups. This suggests that the ingestion of SRP changed the structure of the cecum microbiota. Indeed, the proportions of the phyla *Deferribacteres* and *Verrucomicrobia* detected were significantly lower in the SRP group than in the Ctrl group. Therefore, it was suggested that the supply of SRP to the intestinal tract influenced the microbiota. Since it was considered that the microbiota affects the expression of Reg3γ, a correlation analysis was performed between the abundance ratio of six OTUs that differed between the SRP and Crtl groups and the expression level of Reg3γ. Significant correlations were found for all six OTUs, and a significant correlation coefficient was obtained, especially for *De novo* 59002 (*A. putredinis*), *De novo* 57882 (*F. butyricus*), *De novo* 525 (*M. intestinale*), *De novo* 18129 (*M. intestinale*), and *De novo* 52133 (*M. intestinale*). In the future, further investigation on Reg3γ induction is needed using the type strain of *A. putredinis* and/or *F. butyricus* in a mouse model as well as in *in vitro* cell line experiments. Also of note, the use of enterobacteria for maintaining and improving the health of the host has been gaining much attention; this includes the use of genetically modified microorganisms, which have not been regarded as probiotics. In this context, in addition to the existing lactic acid bacteria and *Bifidobacterium*, a wider range of microorganisms are expected to be used in the future, and these microorganisms are referred to as next-generation probiotics (34). For example, it was reported that *Flavonifractor plautii*, a bacterium isolated from human intestine, improved inflammation (35, 36) and allergic immune responses (37). In addition, in recent studies, genetically modified lactic acid bacteria have been constructed to attenuate cancer (38), inflammation (39, 40), obesity (41), and allergy (42).

To investigate the influence of the microbiota, prediction of the functional potential of the microbiota was performed using PICRUSt. The up-regulation of pathways involved in carbohydrate breakdown was primarily observed. Actually SRP contains 18.5% of carbohydrates, but more than 70% is protein (Table 1). Since the microflora was altered for digestion of SRP, it is necessary to further improve the purity of the protein in SRP and investigate its effect on the intestinal microbiota. It is known that Reg3γ expression is induced not only by the IL-22/STAT3 pathway, but also by stimulation *via* pattern recognition receptors, such as (33). In this context, the up-regulation of the pathway involved in bacterial invasion of epithelial cells suggested that direct stimulation of IECs by the microbiota induced the increase in Reg3γ expression *via* the TLR/MyD88 pathway. Therefore, it was implied that changes in the microbiota due to SRP intake played an important role in the expression of Reg3γ in this study. On the other hand, SRP intake down-regulated the pathway involved in lipopolysaccharide biosynthesis, suggesting that this pathway may be involved in the decrease of the blood LBP concentration.

In conclusion, we conducted a free intake experiment of SRP using mice with the aim of enhancing intestinal homeostasis through the use of functional food components. Ingestion of SRP improved the intestinal barrier function, as seen by a decrease in the blood LBP level, and significantly promoted the expression of Reg3γ, which is known to be involved in the chemical barrier. Since it is known that the intestinal microbiota is involved in the expression of Reg3γ, we analyzed the cecal microbiota, and found a change in β-diversity. In addition, PICRUSt analysis showed that the SRP-diet induced the up-regulation of the functional gene pathway related to the bacterial invasion of epithelial cells. Direct stimulation of the intestinal epithelium *via* the TLR/MyD88 pathway is known to induce the expression of Reg3γ. Therefore, it was suggested that the SRP-induced change in the intestinal microbiota enhanced the intestinal barrier function. In the future, we will investigate the changes in the TLR/MyD88 pathway that result from SRP intake. We hope that SRP can help maintain intestinal homeostasis and contribute to human health.

## Data Availability Statement

The datasets presented in this study can be found in online repositories. The names of the repository/repositories and accession number(s) can be found below: https://www.ddbj.nig.ac.jp/, PRJDB11968.

## Ethics Statement

The animal study was reviewed and approved by Committee for Animal Experiments of Shinshu University.

## Author Contributions

TO, FN, and AM performed the experiments. FN and KU analyzed the microbiota compositions *in silico*. YU and TI supplied the materials. TO, FN, and TS wrote the manuscript. TO and TS designed the research. TS supervised the work. All authors reviewed and approved the final manuscript.

## Conflict of Interest

TI was employed by company Asahimatsu Foods Co., Ltd. The remaining authors declare that the research was conducted in the absence of any commercial or financial relationships that could be construed as a potential conflict of interest.

## Publisher's Note

All claims expressed in this article are solely those of the authors and do not necessarily represent those of their affiliated organizations, or those of the publisher, the editors and the reviewers. Any product that may be evaluated in this article, or claim that may be made by its manufacturer, is not guaranteed or endorsed by the publisher.
